# Motor protein function in skeletal abdominal muscle of cachectic cancer patients

**DOI:** 10.1111/jcmm.12165

**Published:** 2013-11-19

**Authors:** Sultan Taskin, Vera Isabell Stumpf, Jeannine Bachmann, Cornelia Weber, Marc Eric Martignoni, Oliver Friedrich

**Affiliations:** a Institute of Physiology and Pathophysiology, Ruprecht-Karls-UniversityHeidelberg, Germany; b Department of Surgery Klinikum rechts der Isar, Technische Universität MünchenMunich, Germany; c Institute of Medical Biotechnology, Friedrich-Alexander-University Erlangen-NurembergErlangen, Germany

**Keywords:** muscle cachexia, cancer, contractility, myosin isoforms, motility assay

## Abstract

Cachexia presents with ongoing muscle wasting, altering quality of life in cancer patients. Cachexia is a limiting prognostic factor for patient survival and health care costs. Although animal models and human trials have shown mechanisms of motorprotein proteolysis, not much is known about intrinsic changes of muscle functionality in cancer patients suffering from muscle cachexia, and deeper insights into cachexia pathology in humans are needed. To address this question, *rectus abdominis* muscle samples were collected from several surgical control, non-cachectic and cachectic cancer patients and processed for skinned fibre biomechanics, molecular *in vitro* motility assays, myosin isoform protein compositions and quantitative ubiquitin polymer protein analysis. In pre-cachectic and cachectic cancer patient samples, maximum force was significantly compromised compared with controls, but showed an unexpected increase in myofibrillar Ca^2+^ sensitivity consistent with a shift from slow to fast myosin isoform expression seen in SDS-PAGE analysis and *in vitro* motility assays. Force deficit was specific for ‘cancer’, but not linked to presence of cachexia. Interestingly, quantitative ubiquitin immunoassays revealed no major changes in static ubiquitin polymer protein profiles, whether cachexia was present or not and were shown to mirror profiles in control patients. Our study on muscle function in cachectic patients shows that abdominal wall skeletal muscle in cancer cachexia shows signs of weakness that can be partially attributed to intrinsic changes to contractile motorprotein function. On protein levels, static ubiquitin polymeric distributions were unaltered, pointing towards evenly up-regulated ubiquitin protein turnover with respect to ubiquitin conjugation, proteasome degradation and de-ubiquitination.

## Introduction

Cancer cachexia is a severe multi-factorial syndrome associated with loss of lean body mass and adipose tissue. Active hypercatabolism vastly outweighs consequences of starvation, malnutrition or immobility 1. The mechanisms underlying weight loss are highly complex and sustained by chronic inflammation and pro-inflammatory cytokines 2–3. Loss of body weight can be as high as ∼25% in the final months of the patient. In pancreatic cancer, ∼85% of patients become cachectic 1. Most authors define ‘cachexia’ solely by weight loss over time, *i.e*. loss of 10% or more within 6 months 4. Clinically, malnutrition and anorexia result from loss of appetite and altered digestive function, in addition to increased energy expenditure 4–5. A recent international consensus meeting defined new classifications for cancer cachexia as: (*i*) weight loss >5%, including assessment of catabolic drive, muscle mass and strength, (*ii*) systemic inflammation and (*iii*) functional and psychosocial impairment 6. In addition to being a prognostic factor, cancer cachexia also significantly reduces quality of life because of muscle weakness 4,7. Impaired muscle force and early onset of fatigue are present in wasting cancer patients and tumour-bearing animal models 9. Atrophy and wasting in cancer cachexia have been mostly associated with impaired muscle synthesis and increased muscle proteolysis signalling 1,10. On the molecular level, fat atrophy is regulated by tumour-induced lipolysis 12, and a lipid-induced insulin resistance arises through a ‘fat-muscle crosstalk’ that prevents muscle anabolism 12. For catabolic pathways, apart from Ca^2+^-dependent calpain-induced proteolysis of muscle proteins 13,14, the ubiquitin proteasome has been shown to play a dominant role in myofibrillar degradation 16–17, especially in patients with >10% weight loss 1–18. Ubiquitin is a small protein that is linked in ATP-dependent enzymatic reactions to a target protein substrate, thus tagging it for 26S proteasome degradation 1,19. Some studies reported increased ubiquitin mRNA in gastric 21 and pancreatic cancer patients 22 and higher ubiquitin protein levels in tumour-bearing mice 23. However, reported ubiquitin protein levels seem to be completely missing for cachectic cancer patients. This is important, as it is the protein levels that limit functional activity rather than the transcript levels, and transcript/protein dissociations may occur, *e.g*. as a result of post-transcriptional modifications. Ubiquitin exists as a free monomer, as well as conjugated polymers in polyubiquitin chains 24–25. Although some chain types are not linked to cellular functions, others (K48, K63) are known to be used for degradation by the proteasome, NF-κB signalling and DNA repair 26–27. Specifically, proteasome degradation was suggested to be linked to polymer ubiquitin chains of at least four ubiquitin moieties 28. Therefore, one of the present study’s goals was to determine, on the protein level, the distribution of some ubiquitin polymer isoforms in abdominal wall (*rectus abdominis*) muscle samples from cancer patients with assessed cachexia. These were compared with samples from non-cachectic cancer patients as well as from control patients undergoing elective abdominal surgery.

A second goal of this study was to elucidate potential mechanisms of muscle weakness in cancer patients. In animal models of gastrointestinal cancers, an early decrease in skeletal muscle force was regularly observed, *e.g*. in Yoshida AH-130 hepatoma rats 29 and cachectic C-26 adenocarcinoma mice 30. In an experimental Lewis lung cancer animal model of cancer cachexia, force production in *tibialis anterior* muscles was decreased by almost 10% 6 weeks after inoculation, accompanied by a similar loss in muscle mass 31. Thus, absolute maximum force was reduced, while force normalized to muscle mass (specific force) seemed to be unaltered. This argued in favour of muscle weakness being solely associated with muscle wasting but ‘quality’ of cachectic muscle being unaltered 30. With a dynamometer approach, similar results were recently obtained *in vivo* in *quadriceps femoris* muscle from cachectic patients with gastrointestinal cancer compared with healthy volunteers. Force values were reduced ∼40% in cachectic patients; however, cross-sectional area (CSA) normalized muscle strength was unchanged in cachectic tumour patients 32. However, in another study, when looking at the level of single fibres, *tibialis anterior* and *vastus lateralis* samples from a cachectic lung cancer patient produced less than 50% specific force as compared with healthy control participants 8 in addition to the atrophic pattern seen in histology 33. This interesting finding indicated that additional factors at the level of the motorproteins seem to be affected in cachectic muscle. In this aforementioned lung cancer patient, the force loss was explained by a reduction in the Ca^2+^ activation sensitivity of the contractile apparatus, in addition to myosin loss 8. Another very recent study was able to demonstrate the specific force drop to be eminent only in fast, type IIA myosin heavy chain (MHC)-expressing, single *vastus lateralis* fibres from mixed cancer patient moieties 34. Apart from these studies, there are no data available from a larger patient cohort to assess possible cellular mechanisms associated with weakness in cancer cachexia. Our current study was designed as a pilot study similar to the one by Toth *et al*. 34 and also includes cancer patients who either did or did not present with clinical cachexia. However, as controls, we used elective patients with no tumour history to account for similar exposures to inflammatory or stress conditions as in surgical cancer patients instead of omitting controls (*e.g*. in 35) or exploring healthy individuals (as *e.g*. in 34–36).

## Materials and methods

### Patient cohort, muscle sample collection and processing

For biochemistry and physiology tests, muscle samples were collected from *rectus abdominis* muscle of six cachectic and eight non-cachectic tumour patients undergoing explorative or curative abdominal surgery, as well as from five non-tumour patients undergoing elective surgery for other reasons (Table 1). Patients gave written informed consent to participate in the study that took place at the Surgical University Hospital Heidelberg and at the Department of Surgery, Technische Universität München (ethical clearance #301/2001 HD, 1947/07 TUM). Patient inclusion procedures, sample acquisition and preparation, data handling and encryption were performed according to the 1996 Declaration of Helsinki. Most tumour patients included had a firm diagnosis of upper or lower gastro-intestinal tumours (pancreatic cancer, colon cancer, gastric cancer) and were either assigned to a cachectic group (documented involuntary weight loss of >10% within 6 months) or a non-cachectic group (weight loss <5%). This classification of ‘cancer cachexia’ is in agreement with a generally practised clinical classification 4. A recent international classification already classifies weight loss >5% as solid cachexia 6, which includes our cachectic patients. Care was taken to select non-cachectic patients who showed almost no weight loss. According to Fearon *et al*. 6, those patients would be referred to as ‘pre-cachectic’. During surgical procedures (*e.g*. whipple procedure in pancreatic cancer) involving open median laparotomy, care was taken to retrieve tissue that was excised with a sharp blade to minimize damage. A *rectus abdominis* muscle sample was stored in ice-cold Ringer’s solution and transferred to the laboratory, where muscle chunks of 0.3 cm^3^ were dissected and preserved in skinning solution (in mM: Hepes, 40, EGTA, 20, Mg(OH)_2_, 8.82, Na_2_ATP, 8, Na_2_-creatine phosphate, 10, pH 7.0) mixed 1:1 with 99% glycerol and 10 mM DTT (dithiotreitol) at 4°C for 5 hrs before storing at −20°C. This treatment ensured complete permeabilization of cell membranes, yet prevented crystallization at −20°C by glycerol.

**Table 1:** Patient cohorts and diagnoses

Pat.-ID	Tumour	Cachexia	Sex	Age	Diagnosis	BMI	Weight loss (% BW)
ctrl#1	Ø	Ø	♂	76	Kidney donor	28.7	Ø
ctrl#2	Ø	Ø	♀	73	Sigma diverticulosis	26.1	Ø
ctrl#3	Ø	Ø	♀	71	Cholecystolithiasis	22.3	Ø
ctrl#4	Ø	Ø	♂	84	Entero-cutaneous fistula	20	Ø
ctrl#5	Ø	Ø	♀	78	Sigma diverticulosis	21.5	Ø
PnC#1	+	Ø	♂	62	Gastric carcinoma	27.9	Ø
PnC#2	+	Ø	♂	67	Colon carcinoma	24.5	Ø
PnC#3	+	Ø	♀	65	Colon carcinoma	33.3	Ø
PnC#4	+	Ø	♂	67	Colon carcinoma	44.1	Ø
PnC#5	+	Ø	♀	75	Pancreas carcinoma	24.2	Ø
PnC#6	+	Ø	♂	62	Pancreas carcinoma	26.2	Ø
PnC#7	+	Ø	♂	71	Non-Hodgkin lymphoma	25.7	Ø
PnC#8	+	Ø	♀	71	Pancreas carcinoma	23.2	Ø
PC#1	+	+	♀	80	Colon carcinoma	22.2	12.3
PC#2	+	+	♂	74	Lymphoma	26.2	10.6
PC#3	+	+	♀	81	Colon carcinoma	32.0	14.0
PC#4	+	+	♀	42	Pancreas carcinoma	23.2	+ (n.d.)
PC#5	+	+	♂	66	Pancreas carcinoma	20.4	+ (n.d.)
PC#6	+	+	♀	74	Pancreas carcinoma	25.6	12

ctrl: control; PnC: non-cachectic tumour patient; PC: cachectic tumour patient.

### Force transducer recordings

After thawing of muscle samples, fibre bundles of two to three single fibres were manually dissected from the tissue with fine forceps under a stereomicroscope. The fibre bundle was then quickly transferred to a force transducer (KG-7, Scientific Instr., Heidelberg, Germany) and fixed to the stationary and transducer pin. Bundles were adjusted at slack length and sarcomere length was assessed by measuring the interference pattern of a red diode laser (635 nm) through the muscle fibre bundle. The preparation was immersed in ‘internal solution’ with defined pCa values between 9 and 4.3, which were obtained by appropriate mixtures of high activating HA (in mM: Hepes, 30, EGTA, 30, CaCO_3_, 30, Mg(OH)_2_, 7.46, Na_2_-creatine phosphate, 10, Na_2_ATP, 8, KOH, 66; pH 7.0) and high relaxing HR (same as HA, but with 8.1 mM Mg(OH)_2_ and omitting CaCO_3_) solutions. At each pCa value, steady-state force-pCa values were established 37. The permeabilization of all membranes ensured complete diffusional control over the myoplasm and the contractile apparatus, so that the Ca^2+^ concentrations at the site of the myofibrillar proteins could be faithfully ‘clamped’ to the pCa of the bathing solutions. pCa-force relationships from each individual recording were fitted with a Hill function yielding the flexion point, pCa_50_, and the steepness h. The first parameter, pCa_50_, represents a measure for the Ca^2+^ sensitivity of the contractile apparatus, the second parameter the cooperativity of the Ca^2+^-troponin C binding. Absolute maximum force values were evaluated as the difference in force at pCa of 9 and 4.3 and were computed for each patient. Cohort values were analysed using a one-way anova with post-hoc test (Dunn’s method).

### *In vitro* motility assays of single muscle fibre myosin extracts

*In vitro* motility assays from single fibre myosin extracts were performed as previously described with a flow cell 39–40. A more detailed description is given in Data S1.

### SDS-PAGE analysis of myosin isoform distribution in patient samples

Myosin heavy chain (MHC) and light chain (MLC) electrophoresis was performed as described previously 38–44. A more detailed description is given in Data S1.

### Quantitative ubiquitin multimer protein analysis in patient muscle samples

Quantitative protein analysis from ubiquitin immunoblots was performed with an approach similar to that given in Mollica *et al*. 45. Purified ubiquitin standards were obtained as described 46. A more detailed description is given in Data S1.

## Results

### Ca^2+^ sensitivity of the contractile apparatus is increased in *rectus abdominis* muscle from cachectic cancer patients

**Figure 1:**
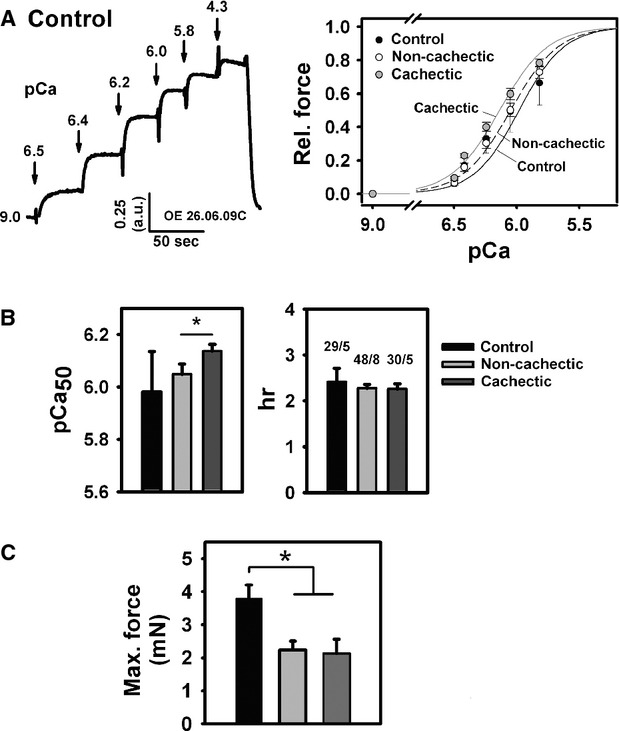
Ca^2+^ sensitivity of the contractile apparatus and maximum force values from *rectus abdominis* muscle biopsies in control, non-cachectic and cachectic cancer patients. (A) Representative recording of force response to increasing Ca^2+^ concentrations (decreasing pCa) in one control patient sample (left panel; ordinate—relative force units; abscissa—time). Right panel shows the mean pCa-force curves reconstructed from group data and fitted with a sigmoidal fit. For a more quantitative analysis, steady-state force relations from each patient within a group were fitted with a Hill function and pCa_50_ and Hill coefficient extracted. (B) Mean values of pCa_50_ indicate a significantly increased Ca^2+^ sensitivity of the contractile apparatus in cachectic *versus* non-cachectic patients, while Hill coefficients h were similar. (C) Maximum absolute force was significantly compromised in samples from cancer patients, unrelated to the presence of cachexia. n/m indicates number of fibre bundles n studied in m participants within each group and are the same in (B) and (C). *P < 0.05.

Figure 1A shows a representative original recording of force response from a small bundle of control patient (ctrl#1) muscle fibres exposed to various pCa. A typical staircase pattern can be seen. The mean relative force-pCa relations for each patient population suggest a left-shift in cachectic tumour patient samples towards larger pCa values. This is quantified by the mean pCa_50_ values and Hill coefficients from many recordings in samples from five control, eight non-cachectic and five cachectic tumour patients (Fig. 1B). Ca^2+^ sensitivity of the contractile apparatus was significantly increased in cachectic over non-cachectic patients. Values for each individual patient are given in Table 2. Absolute force values assessed at pCa of 4.3 were 3.8 ± 0.4 mN in control patients, 2.2 ± 0.3 mN in non-cachectic and 2.1 ± 0.4 mN in cachectic patients and showed a significant decrease in cancer patients, whether they were cachectic or not (Fig. 1C). This indicates that samples from ‘pre-cachectic’ patients already share the same degree of weakness as from cachectic patients, at least at the level of small multi-cellular preparations.

**Table 2:** pCa-force results (pCa50, h) for each individual patient sample

Pat.-ID	pCa50	h	*n*	SL (μm)
ctrl#1	6.21 ± 0.02	2.69 ± 0.34	6	2.1
ctrl#2	6.23 ± 0.07	2.76 ± 0.42	6	2.5
ctrl#3	6.14 ± 0.02	3.18 ± 0.41	6	2.1
ctrl#4	5.91 ± 0.02	1.65 ± 0.13	6	2.4
ctrl#5	5.40 ± 0.02	1.78 ± 0.09	5	2.3
PnC#1	6.01 ± 0.01	2.42 ± 0.16	6	2.3
PnC#2	6.19 ± 0.03	2.33 ± 0.34	6	2.4
PnC#3	6.13 ± 0.03	2.23 ± 0.45	6	2.4
PnC#4	6.07 ± 0.02	2.62 ± 0.22	6	2.7
PnC#5	5.98 ± 0.03	2.37 ± 0.27	6	2.2
PnC#6	6.11 ± 0.02	2.08 ± 0.21	6	2.2
PnC#7	6.03 ± 0.02	2.32 ± 0.21	6	2.1
PnC#8	5.83 ± 0.02	1.87 ± 0.01	6	2.3
PC#1	6.06 ± 0.02	2.32 ± 0.25	6	2.1
PC#2	6.16 ± 0.03	2.35 ± 0.32	6	2.2
PC#3	6.23 ± 0.03	2.60 ± 0.34	5	2.2
PC#4	6.13 ± 0.02	2.06 ± 0.19	7	2.5
PC#5	6.10 ± 0.03	1.97 ± 0.21	6	2.4
PC#6	5.48 ± 0.02§	1.88 ± 0.09	4	2.3

§ Significant outlier (*P* < 0.05).

SL: sarcomere length; n: number of recordings from different fibre bundles of each patient sample.

### *In vitro* motility recordings in *rectus abdominis* single cell extracts show a shift towards faster sliding velocities

**Figure 2:**
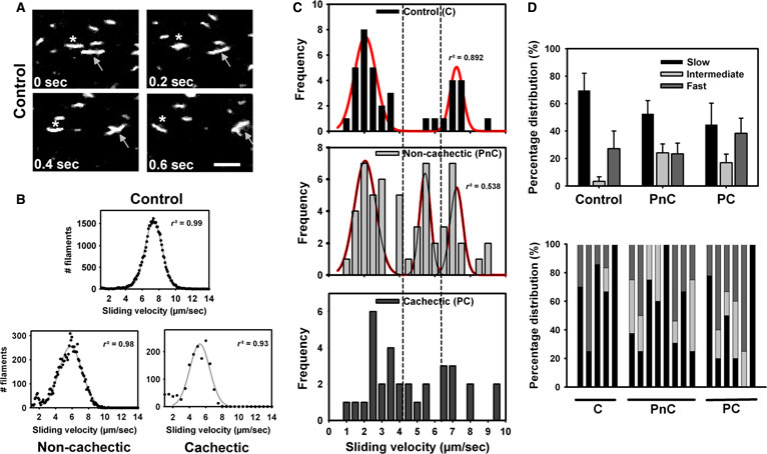
*In vitro* motility assays suggest a shift of slow to intermediate sliding velocities in cancer patients. (A) Representative images from actin-filament sliding sequences of a control patient single cell extract. Two individual filaments are tracked in time (arrow, asterisk). Velocity distributions for each experiment were assessed and fitted using a Gaussian curve to extract median velocities (B). (C) Median sliding velocities for each patient group revealed a clear double-peak histogram distribution in control participants, but a triple-peak velocity distribution in non-cachectic cancer patients. A triple band for velocity was empirically defined according to maximized correlation coefficients of the Gaussian multi-peak fits. This gave a cut-off of <4.25 μm/sec. for slow velocities, intermediate velocities ranged between 4.25 μm/sec. and 6.25 μm/sec. and >6.25 μm/sec. for fast sliding velocities. (D) For each patient, percentage distribution of filament velocities in each velocity group shows substantial scattering (lower panel). Almost no intermediate filament velocities were found in control patients, while there was a tendency towards higher intermediate filament velocity percentages in cancer patients, mainly because of a reduction in slow filaments (upper panel).

To assess the molecular interaction of patient motorprotein function, single cell myosin extract *in vitro* motility assays were performed. Figure 2A shows example images from an image sequence performed in a single cell myosin extract from a control patient, demonstrating the sliding of fluorescently labelled actin filaments over patient myosin heads. Applying image segmentation and filament tracking of individual actin filaments (*e.g*. marked with an arrow and asterisk in Fig. 2A), filament velocity distributions from hundreds of filaments observed in a single video sequence were obtained and fitted with a Gaussian distribution (Fig. 2B, showing results from one such experiment for one patient in each group). Collecting mean sliding velocities in 0.5 μm/sec. bins identified a multi-peak distribution, where a slow (<4.25 μm/sec.), an intermediate (4.25–6.25 μm/sec.) and a fast (>6.25 μm/sec.) sliding velocity distribution was assessed. Interestingly, control patients produced almost no filaments with intermediate sliding velocities (Fig. 2C and D), whereas tumour patients produced a much larger proportion of intermediate filaments with a reduction in slow filaments. Values for each individual patient are given in Figure 2D and Table S1.

### SDS-PAGE analysis of *rectus abdominis* myosin extracts from small muscle fibre bundles suggests a shift towards faster isoforms in cancer cachexia

**Figure 3:**
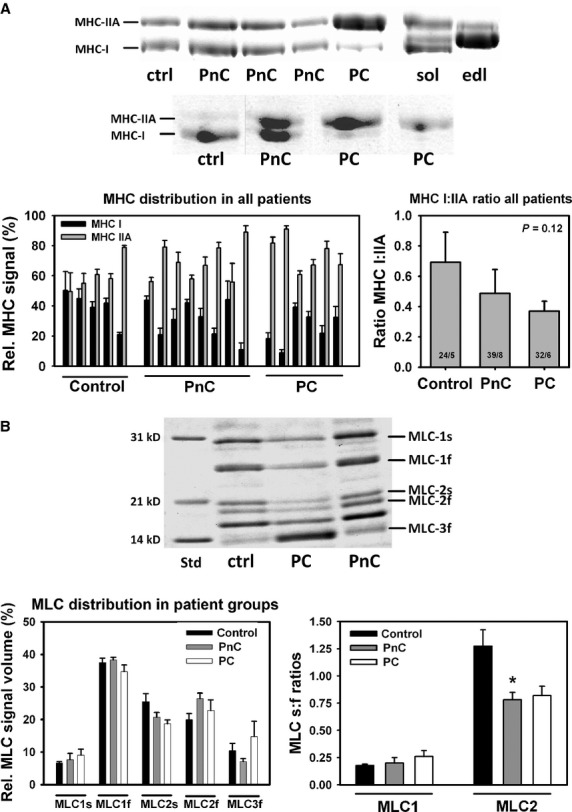
SDS-PAGE analysis of motorprotein distribution suggests a shift in heavy chain distribution towards faster isoforms in cachectic cancer patients. (A) Two MHC gels from *rectus abdominis* muscle samples from control (ctrl), non-cachectic (PnC) and cachectic cancer patients (PC) are shown with a sample from murine *soleus* (sol) and *extensor digitorum longus* (edl) muscles, for which isoform distribution is well known. Although there is some scattering between patients regarding relative contents of MHC-I and MHC-IIA signals, there is a consistently larger fraction of faster MHC-IIA in the PC group. This is also reflected by a largely reduced MHC I:IIA ratio, which declines from control to non-cachectic and cachectic patients. Myosin light chains MLC isoforms were analysed from gels such as shown in (B). Apart from a significant smaller fraction of MLC-2s over MLC-2f in cancer patients over controls, this was not significant in cachectic patients and there was no major difference in the other isoforms (*P < 0.05 *versus* control).

Figure 3A shows two representative MHC gels from individual control (ctrl), non-cachectic (PnC) and cachectic (PC) tumour patients as well as a sample from murine *soleus* (sol) and *extensor digitorum longus* (edl) muscle to define MHC-I and MHC-IIA bands. Relative protein amounts evaluated from multiple gels for each patient are given in Figure 3A. There was a tendency for smaller MHC I:IIA ratios in cachectic patients over non-cachectic and control patients. For MLC (Fig. 3B), types 1, 2 and 3 were detected with their respective slow and/or fast isoforms by their molecular weights according to published values. Although the prominent band below MLC-2f may be suggestive of the MLC-3s isoform, it was not included in the further analysis as it could not unambiguously be identified (see 47–48). The data suggest somewhat larger s:f ratios for MLC-1 and smaller s:f ratios for MLC-2 for tumour patients when compared with control patients, although this was not significant for the cachexia group, but was significant for the pre-cachectic group in case of MLC-2 (*P* < 0.05, one-way anova and Dunn’s post-hoc analysis).

### Ubiquitin isoform distributions are unaltered in *rectus abdominis* samples from tumour patients regardless of the presence or absence of cachexia

**Figure 4:**
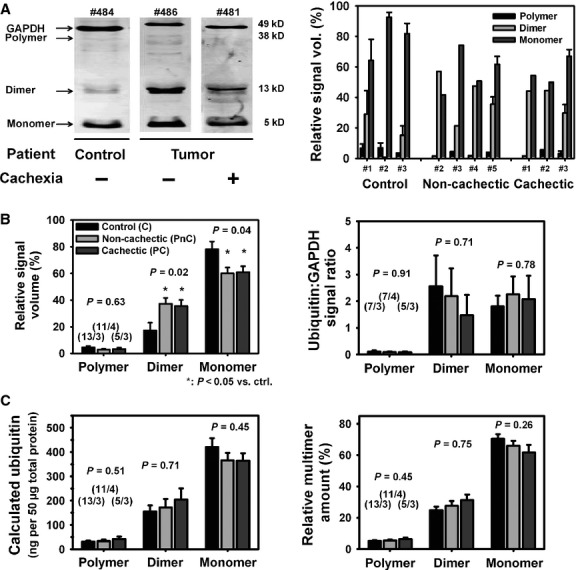
Quantitative ubiquitin Western blot analysis indicates similar static protein amounts of some ubiquitin multimer levels in abdominal muscle from control, non-cachectic and cachectic cancer patients. (A) Representative Ubiquitin Western blot of *rectus abdominis* muscle samples from control, non-cachectic and cachectic tumour patients (left panel), as well as relative signal volume densities from individual patients (right panel). Volume densities on their own suggest vastly enlarged dimer fractions and significantly reduced monomer fractions in tumour patients over controls, an observation which is no longer present when normalizing ubiquitin to GAPDH (B). With the slopes from the calibration curves for multimers (Fig. S1), multimer protein weights and their relative distribution were quantified (C). Patient identifiers in (A) are: #484 = ctrl#3; #486 = PnC#4; #481 = PC#2.

Tumour cachexia has been widely associated with hypercatabolism of muscle proteins through proteolytic pathways, *i.e*. the ubiquitin machinery 1–49, although different muscles are differentially affected 18. For *rectus abdominis* muscle in cancer cachexia patients, only mRNA levels for ubiquitin are available 21. As qualitative assessment of total protein levels can be prone to misinterpretations 45, we sought to perform a quantitative determination of absolute ubiquitin protein isomer distributions in our *rectus abdominis* patient samples. Figure S1 shows representative Western blot results of our calibration procedure using purified ubiquitin separated as monomers, dimers and polymer fractions (see Discussion). Also shown is the densitometric analysis for either alkaline phosphatase (AP) or a fluorescence-based technology. The AP-based calibration followed a linear relationship of signal volume with ubiquitin mass, while the calibration was exponential for fluorescence detection. For both methods, the calibration curves were exclusively used in subsequent calculations of patient sample ubiquitin amounts. Note that we refer to the polymer level not explicitly as a fixed polyubiquitin moiety, but rather as ‘polymer’, as this band may comprise a mixture of higher ubiquitinated chains (∼47 kD reflecting an average of six to eight ubiquitin chains at a molecular weight of ∼6–8 kD). Figure 4A shows representative Western blots from a control patient, a non-cachectic and cachectic tumour patient biopsy simultaneously stained for ubiquitin and glyceraldehyde-3-phosphate dehydrogenase (GAPDH). In addition, mean relative polyubiquitin distributions from all individual patients are shown. When analysing the mean relative signal densities for each patient cohort, a significant shift of ubiquitin distribution towards more dimers and less monomers in tumour patients over control patients was noted. However, normalization to a housekeeping protein (GAPDH) revoked this difference to similar values in all groups (Fig. 4B). This was also reflected in our quantitative ubiquitin assessment by comparable absolute amounts of ubiquitin monomers, dimers and polymers (Fig. 4C). Therefore, our quantitative approach is validated by an ‘internal’ GAPDH normalization standard.

## Discussion

### Contractility in cachectic human muscle samples

Although determinants of muscle performance were previously described in cancer patients 34,35, few studies focus on cachexia as a main determinant. The common concept that weakness in cancer cachexia was predominantly determined by cachexia-associated atrophy 32–33 has been extended by some recent pilot studies that demonstrated that additional factors apart from atrophy, *i.e*. at the motorprotein levels, must contribute to a drop in the ‘quality of muscle force’ 8–34. Most of those studies, however, were either small case pilot studies involving single individuals 8, compared cachectic and/or non-cachectic cancer patients with healthy volunteers 33,34 or involved cachexia patients with a mixed underlying disease background 33. This prompted us to collect muscle biopsies from several cachectic cancer patients for single fibre and motorprotein studies of contractility. We also used two additional patient cohorts that probably better define the independent variables ‘cachexia’ and ‘control’: (*i*) cancer patients who did not develop a cachexia over 6 months (‘pre-cachectic’ according to 6) and (*ii*) non-tumour patients undergoing elective surgery. This ‘control’ group (no tumour, elective surgery) may be of importance as, compared with healthy individuals, patients undergoing surgery *per se* present with an elevated state of stress-related inflammatory responses 50–51. Using this study design, maximum force values at a pCa of 4.3 were significantly compromised in our samples from cancer patients when compared with control patients. This was regardless to cachexia being present or not. During short tetanic contractions, Ca^2+^ levels in mammalian muscle peak at around 20–25 μM at 28°C 52. Therefore, our values taken at pCa of 4.2 (corresponding to ∼45 μM free Ca^2+^) reflect a Ca^2+^-saturated maximum force that can be compared between patient groups. As we primarily focused on the contractile apparatus contributing to the force in cachexia and not the atrophy aspect, cross-sectional areas of the fibre bundle preparations were not assessed. However, we took care to assess a similar number of fibres within every bundle prepared from each patient specimen. In a previous study, specific force in muscle samples obtained from one tumour patient was also found to be reduced 8–33. This also applied to a very recent pilot study in mixed cachectic and non-cachectic cancer patients, where single fibre *vastus lateralis* muscle maximal Ca^2+^ activated specific tension was unaltered in MHC-I expressing fibres, but ∼15% reduced in MHC-IIA fibres 34. Force loss was specific for ‘cancer’, but not for ‘cachexia’, similar to our findings in *rectus abdominis* muscle. In their study, Toth *et al*. 34 found no differences in myosin or actin protein contents in any of the groups (control, cachectic and non-cachectic cancer patients), challenging previous concepts of selective myosin protein loss and pure atrophy being responsible for cancer-related weakness, but favouring cellular and molecular mechanisms of single fibre–related weakness instead. For example, one of their major outcomes was that single fibre tension in MHC-IIA expressing fibres was reduced in limb muscle from cancer patients because of a reduction in the number of strongly bound cross-bridges and cross-bridge stiffness 34.

One unexpected finding of our study was a left-shift of the pCa-force curve in cachectic patient samples with significantly larger pCa_50_ values in cachectic over non-cachectic patients. This indicates an increased steady-state Ca^2+^ sensitivity of the contractile apparatus in cachexia that could indicate a shift in myosin isoform distribution in cachectic muscle. In human *vastus lateralis* muscle biopsies, fast MHC isoforms (type IIA, IIB) have significantly larger pCa_50_ values and normalized force than slow type I isoforms 53. In contrast, in *medial gastrocnemius* muscle from monkeys, Ca^2+^ sensitivity and MHC isoforms had reverse relationships 54. Although no preferential target of myosin proteolysis towards MHC I or MHC II isoforms was detected in *tibialis anterior* and *vastus lateralis* samples from cancer and critical illness patients with substantial muscle atrophy and myosin loss 8,33, altered myosin expression in skeletal muscle (*soleus*,*gastrocnemius*) of tumour-bearing mice from MHC I to MHC II (slow-to-fast transition) was found 55. This is also seen in our patient cohorts by two independent observations: *in vitro* motility assays and SDS-PAGE analysis. In the latter, there was a progressive decline in overall MHC IIA expression over MHC I from control to non-cachectic and cachectic patients. Although this was not yet significant, it marks an important trend that should be substantiated in future trials involving larger patient numbers. The preferential increase in MHC II fits with the increased myofibrillar Ca^2+^ sensitivity seen in human skeletal muscles 53. Ochala & Larsson 8 observed overall reduced myofibrillar Ca^2+^ sensitivity in their patient population. However, this population represented a mixture of several critically ill patients and one single cancer patient. A second independent method that substantiates our hypothesis is reflected by the *in vitro* motility assay results. These are, to our knowledge, the first recordings of molecular motorprotein interaction in human cachectic muscle. The velocity distributions allowed an empirical classification into three distinct bins: a slow myosin isoform, a fast isoform and an intermediate isoform. The peaks of the frequency distributions for the slow and fast isoforms are in good agreement with previously described values for different slow- and fast-twitch muscles 38–40. The actomyosin complex, with a slower sliding velocity compared with the fast bin (intermediate), may probably be explained by potential differences in light chain phosphorylation states. This can be assumed, as the MHC distribution seen in the SDS-PAGE was still dichotomic with only MHC I and MHC II bands. MLC phosphorylation slows down the sliding velocities in motility assays within the same MHC regime 56. Interestingly, the percentage distributions of sliding velocities showed a tendency for decreased slow and increased fast values in control over non-cachectic and especially cachectic cancer patients. Although not statistically significant, this would fit with the tendency for increased MHC II and decreased MHC I expression in our SDS-PAGE analysis. Toth *et al*. 34 did not observe any significant shifts in MHC isoform expression in *vastus lateralis* muscle homogenates from cancer patients compared with controls; however, their results are compatible with ours, with slightly reduced MHC I and increased MHC II (see their Fig. 3). Nevertheless, their primary findings provide a very elegant explanation model of compromised force unrelated to muscle wasting in cancer patients, *i.e*. altered cross-bridge properties with reduced number of strongly bound cross-bridges and stiffness. It may be likely that a combination of the MHC isoform distribution observed by us and the alteration in cross-bridge properties 34 may overcome our observed increased myofibrillar Ca^2+^ sensitivity to produce single fibre weakness. Therefore, our study, which is the second study to evaluate the effects of cancer on skeletal muscle function in humans at the cellular and molecular level, is complementary to the one of Toth *et al*. 34.

### Quantitative ubiquitin protein analysis in cachectic human muscle samples

Muscle protein degradation through the ubiquitin proteasome pathway is the major contributor to muscle wasting in cancer cachexia, associated with myosin proteolysis and weakness. However, reports vary for different tumour types. In *gastrocnemius* muscle of MAC16 adenocarcinoma mice, there was a good correlation of expression of ubiquitin-conjugating enzyme E2_14k_, proteasome subunit C2 mRNA and protein levels with weight loss 18. Proteasome-specific tyrosine release (indicator for protein degradation) was at a maximum at ∼20% weight loss and decreased for more severe cachexia 18. Translated to our patient collective, this would correspond to near-maximum proteasome activation between 10% and 15% weight loss. However, a direct comparison with our study has to be taken with caution, as tyrosine release results for human muscles are elusive. Direct measurements of ubiquitin mRNA in *rectus abdominis* muscle biopsies from gastric cancer patients showed an approximate 2.5-fold increase in ubiquitin transcripts, but protein levels were not given 21. In contrast, in small cell lung cancer patients in the pre-cachexia stage, no change in ubiquitin 26S proteasome activity or E3 ligase mRNA expression was detected compared with healthy control cases. It was concluded that local muscle inflammation was required to initiate ubiquitin-dependent muscle wasting in cancer 57. Interestingly, ubiquitin polymer protein distributions in human muscle in cancer cachexia have not yet been reported. This is important as the polyubiquitination of target substrates precedes the proteolytic degradation by the 26S proteasome 19, suggesting that polymers may drive degradation more efficiently than monomers. Indeed, a minimum of at least four ubiquitin moieties was shown to be required to be recognized by the 26S proteasome 28. Also, as local inflammation was postulated to initiate proteasome degradation in cachectic muscle 57, we reasoned that healthy control cases might not reflect a proper control group for cancer cachexia. We, therefore, chose non-cancer elective surgery patients as controls. When analysing protein densities of the ubiquitin isomers, a significant increase in dimer distribution at the cost of monomers was seen in *rectus abdominis* samples from cancer patients compared with surgical controls, regardless of whether they were cachectic or not (Fig. 4B). However, when normalized to GAPDH, this effect was abolished. This was also confirmed by our quantitative immunoblot protein analysis approach using an adequate calibration technique 45. Our values of ∼200 ng/50 μg total protein for dimers and ∼400 ng/50 μg for monomers are well in the range for values of 2–3 μg free ubiquitin per mg total protein given in a recent rat muscle trauma model 58. Thus, we are confident that our technique faithfully tracks ubiquitin protein multimers in skeletal human muscle. It has to be noted that, apart from our monomer and dimer signals, we refer to our ∼47 kD band as ‘polymers’. Given values for ubiquitin monomers of ∼8 kD 59 and our somewhat lower detection at ∼6 kD, our ‘polymer’ signal most likely comprises polyubiquitinated protein chains of six to eight ubiquitin molecules. Given the fact that the polyubiquitin degradation signal must comprise at least four ubiquitin moieties 28, the evaluation of the monomers and dimers is probably less relevant when compared with the polymer bands in our cachectic patient samples. Nevertheless, the unchanged absolute protein levels do not indicate a non-up-regulated ubiquitin proteolysis, but rather reflect a static index. This is because the amount of ubiquitin conjugates depends on the equilibrium between the rate of conjugation to the substrates, their rate of degradation by the proteasome and their rate of de-ubiquitination. As all processes are increased in catabolic states, including cancer cachexia, the only conclusion that can be drawn from our ubiquitin analysis is that all processes seem to be equally up-regulated. Similar results, *i.e*. no accumulation of ubiquitin polymers, were found in sepsis-induced muscle proteolysis 60.

In summary, our study provides new and unexpected insights into muscle function in cancer cachexia patients on the cellular and subcellular level that are complementary to the study of Toth *et al*. 34. The changes seen in force production and Ca^2+^ sensitivity are probably explained by altered expression of myosin isoforms. Also, ubiquitin protein profiling is probably not a sensitive parameter to detect the well-known up-regulation of the ubiquitin proteasome. Future trials with larger patient numbers are needed to further address this point.

## Supporting information

**Figure S1.** Quantitative calibration of ubiquitin polymer levels with a ubiquitin protein standard purified from erythrocytes. **Table S1.** Filament distribution—with an in vitro motility assay—derived slow, intermediate and fast filaments for each individual patient sample investigated. **Data S1.** Materials and methods.
