# Supplementary material for: Motor protein function in skeletal abdominal muscle of cachectic cancer patients
Source: J Cell Mol Med. 2013 Nov 19;18(1):69–79. doi: 10.1111/jcmm.12165 (PMC3916119; doi:10.1111/jcmm.12165)
Supplement: Supplementary file 1 — Figure S1. Quantitative calibration of ubiquitin polymer levels with a ubiquitin protein standard purified from erythrocytes. Table S1. Filament distribution—with an in vitro motility assay—derived slow, intermediate and fast filaments for each individual patient sample investigated. Data S1. Materials and methods. [file jcmm0018-0069-sd1.doc]

**Supplemental Material and Methods:**

**In vitro motility assays of single muscle fibre myosin extracts.** Single fibres were dissected from thawed patient muscle samples and flushed with myosin extraction buffer (mM: KCl, 300, Hepes, 25, MgCl_2_, 4, EGTA, 4, Na_2_ATP, 2, DTT, 10, pH 7.6) on a nitrocellulose-coated coverslip within a flow-cell for 20 min [39]. Following several washing steps, rhodamin-phalloidin labelled purified rabbit skeletal muscle actin was added to the chamber and allowed to bind to the coverslip coated myosin heads of patient-extracted myosin. After a ~2 min incubation, unbound F-actin was washed off and the remaining preparation incubated in a ‘scavenger-buffer’ solution (containing in mM: Hepes, 50, KCl, 50, MgCl_2_, 8, EGTA, 10, DTT, 20, glucose, 10, as well as BSA, 0.05 w/v %, glucose oxidase 0.01 w/v % and catalase 0.002 w/v %) to minimize photobleaching. The coverslip was mounted on the stage of an inverted epifluorescence microscope (Olympus, IX-70, Hamburg, Germany) equipped with a CCD camera (PCO Pixelfly, PCO, Germany). Fluorescence was excited at ~545 nm and emission collected at >570 nm. After addition of ATP to the chamber, crossbridge cycling was initiated as visualized by sliding fluorescent filaments that were recorded at 10 fps. For each single fibre myosin extract, an average of four to six videos was recorded. Several single fibre extracts were run for each patient sample. Every image stack was analyzed using a custom-made segmentation and filament tracking algorithm [40]. The imaging algorithm evaluated the velocity distribution of all filaments in the video sequence that is presented as a histogram and fitted with a Gaussian curve to extract the median velocity of each experiment. For each patient sample, the mean velocity and SEM from several recordings were assessed and compared among patient groups (one-way ANOVA with post-hoc testing, Bonferroni or Holm-Sidak).

**SDS PAGE analysis of myosin isoform distribution in patient samples.** For MHC separation, a 10 % SDS gel [41] was used (75 V 4 mA at 4 °C for 24-27 hr); MLC were run on a 12 % gel (300 V, 15 mA, room temperature for ~90 min). For each lane, approximately 15 single fibres were collected from a single patient sample. Myosin was extracted for 25 min at room temperature in protein extraction buffer, centrifuged, mixed 1:2 with standard protein buffer and loaded onto the running gel [38]. Both standard Coomassie blue or silver stainings were performed and gels scanned and analyzed for background corrected volume densitometry using LabImage gel and Western blot analysis software (Kapelan Bio-Imaging GmbH, Leipzig, Germany) and ImageJ (NIH software; <http://rsbweb.nih.gov/ij/>). Broad range protein standards were from peqLab (peqGold prestained protein marker IV, peqLab Erlangen, Germany) or Anamed Elektrophorese GmbH (Gross-Bieberau, Germany). For classification of MHC isoforms, samples from mouse *edl* (MHC IIB, IIX) and *soleus* muscle (MHC I, IIA) were also run on the same gels as an internal standard. Myosin light chain isoforms were identified according to documented molecular weights [42‑44]: MLC-1s, ~27 kDa, MLC-1f, ~25 kDa, MLC-2s, ~19 kDa, MLC-2f, ~18 kDa, MLC-3f, ~16 kDa. For each lane, MHC distribution in *rectus abdominis* muscle samples was analyzed and represented as relative MHC-I and MHC-IIA percentage with respect to total MHC. MLC-1s/f, MLC-2s/f, MLC-3f are presented in the same way. Each patient sample was run at least in duplicate. Data for each individual patient are given, as well as pooled data for the control, non-chachectic and cachectic cancer patients. Patient groups were compared using a one-way ANOVA with post-hoc analysis.

**Quantitative ubiquitin multimer protein analysis in patient muscle samples.** Similar to the myosin separation, ~15 single fibres from each patient sample were incubated in protein extraction buffer, processed, separated on a 15 % SDS gel (~0.2 mA for 1.5 hr at RT) and then blotted to a PVDF membrane using the standard semi-dry Western blotting technique. After protein transfer, membranes were blocked for 1-2 hr with 2.5 % milk powder or 0.3 % bovine serum albumin (BSA) in PBS, washed and then incubated overnight at 4 °C with primary antibodies against ubiquitin (1:400 mouse monoclonal AB, Ub-P4D1; Santa Cruz sc-8017, Heidelberg, Germany) and GAPDH (1:40.000; glyceraldehydes-3-phosphate dehydrogenase; Santa Cruz), a commonly used housekeeping protein. After washing three times with TBS-Tween (10 mM Tric-HCl, 150 mM NaCl, 0.1 % Tween20, pH 7.4), membranes were incubated with secondary antibodies (polyclonal rabbit anti mouse IgG) conjugated either to alkaline phosphatase (AP) or Alexa-Fluor 633. All membranes were handled identically for AP reaction procedure, and after stopping the reaction, membranes were scanned and analyzed using LabImage software. For experiments involving fluorescent secondary-antibody detection, membranes were scanned using the Li-Cor Odyssey infrared imaging system (Li-Cor Biosciences, Germany). Volume densities were background corrected and are presented as absolute and relative values as well as GAPDH:ubiquitin ratios. For ubiquitin signals, three prominent bands representing monomers, dimers and polymers (i.e. hexamers to octamers) were detected. Western blot results can be prone to misinterpretation if only density values or housekeeping ratios are involved. This is particularly true when protein densities lie outside a region of density (signal-protein amount) linearity. Therefore, we followed the approach of Mollica et al. [45] and used a quantitative calibration of ubiquitin signals to quantify absolute protein amounts, from which number of molecules can even be calculated. For this, a standard consisting of highly purified protein must be established [45]. We modified a high-affinity ubiquitin purification protocol that yields ~75 % of the ubiquitin available in human red blood cells [46]. Briefly, red blood cells from expired packed cell units, kindly provided by the local blood bank, were centrifuged, haemolysed and treated with 5 % perchloric acid (PCA). The lysate was centrifuged at 5,000 rpm for 10 min, incubated with 5 % trichloric acid (TCA) and ubiquitin allowed to precipitate overnight at 4 °C. The precipitate was then spun-down, the pellet resuspended in 1.5 M lower Tris buffer (pH 8.8), centrifuged, the supernatant boiled, recentrifuged and the resulting supernatant freeze-stored in aliquots. Using Bradford technique, we estimated a yield of ~150 µg ubiquitin per g of haemoglobin. Western blotting of purified ubiquitin confirmed its segregation into monomers, dimers and ‘polymers’ (according to [24]). Using known amounts of total ubiquitin protein input, a calibration curve was obtained where increasing amounts of protein corresponded to increasing volume signals for each ubiquitin multimer. The relative multimer distribution for each known total ubiquitin amount was assessed and used as input for the calculation of the total ubiquitin amount in each patient sample, as well as the projected individual amounts of monomer, dimer and polymer. This calculation was possible since the separation percentage was relatively stable regardless of the amount of total ubiquitin in the calibration curve (not shown).

**Supplemental Table 1: Filament distribution - using an in vitro motility assay - derived slow, intermediate and fast filaments for each individual patient sample investigated.**

| **Pat.-ID** | **median velocity (µm/s)** | | |
| --- | --- | --- | --- |
|  | **slow (<4.25)** | **intermediate (4.25 – 6.25)** | **fast (>6.25)** |
|  |  |  |  |
| **ctrl#1** | 70 % | 0 % | 30 % |
| **ctrl#2** | 25 % | 0 % | 75 % |
| **ctrl#3** | 86 % | 0 % | 14 % |
| **ctrl#4** | 67 % | 16 % | 16 % |
| **ctrl#5** | 100 % | 0 % | 0 % |
|  |  |  |  |
| **PnC#1** | 37 % | 37 % | 25 % |
| **PnC#2** | 25 % | 25 % | 50 % |
| **PnC#3** | 75 % | 25 % | 0 % |
| **PnC#4** | 60 % | 40 % | 0 % |
| **PnC#5** | 100 % | 0 % | 0 % |
| **PnC#6** | 31 % | 15 % | 54 % |
| **PnC#7** | 67 % | 0 % | 33 % |
| **PnC#8** | 25 % | 50 % | 25 % |
|  |  |  |  |
| **PC#1** | 78 % | 0 % | 22 % |
| **PC#2** | 20 % | 20 % | 60 % |
| **PC#3** | 50 % | 17 % | 33 % |
| **PC#4** | 20 % | 40 % | 40 % |
| **PC#5** | 0 % | 25 % | 75 % |
| **PC#6** | 100 % | 0 % | 0 % |

**Supplemental Figure 1:**

**Suppl. Fig.1: Quantitative calibration of ubiquitin polymer levels using a ubiquitin protein standard purified from erythrocytes.** Western blot using an AP-coupled antibody to detect increasing amounts of ubiquitin for calibration using purified ubiquitin (see Methods). Monomers (~4-6 kDa), dimers (~10-14 kDa) and some higher polymers can be clearly distinguished. Using known amounts of ubiquitin total protein input, calibration curves for the densitometric signal volume of each multimer was established.
